# 14, 15-EET induces breast cancer cell EMT and cisplatin resistance by up-regulating integrin αvβ3 and activating FAK/PI3K/AKT signaling

**DOI:** 10.1186/s13046-018-0694-6

**Published:** 2018-02-09

**Authors:** Jing Luo, Jian-Feng Yao, Xiao-Fei Deng, Xiao-Dan Zheng, Min Jia, Yue-Qin Wang, Yan Huang, Jian-Hua Zhu

**Affiliations:** 1 Department of Immunology, Tongji Medical College, Huazhong University of Science and Technology (HUST), Wuhan, Hubei People’s Republic of China; 2 Laboratory of Clinical Immunology, Wuhan No.1 Hospital, Tongji Medical College, Huazhong University of Science and Technology (HUST), 215 Zhongshan Dadao, Wuhan, Hubei 430022 People’s Republic of China; 3 Quanzhou Maternal and Child Health Care Hospital, Quanzhou, People’s Republic of China; 4 Department of Radiology, Tongji Hospital, Tongji Medical College, Huazhong University of Science and Technology (HUST), Wuhan, Hubei People’s Republic of China

**Keywords:** Breast cancer, 14, 15-EET, EMT, Cisplatin resistance, αvβ3/FAK/PI3K/AKT signaling

## Abstract

**Background:**

14,15-epoxyeicosatrienoic acid (14,15-EET) is an important lipid signaling molecule involved in the regulation of tumor metastasis, however, the role and molecular mechanisms of 14,15-EET activity in breast cancer cell epithelial-mesenchymal transition (EMT) and drug resistance remain enigmatic.

**Methods:**

The 14, 15-EET level in serum and in tumor or non-cancerous tissue from breast cancer patients was measured by ELISA. qRT-PCR and western blot analyses were used to examine expression of integrin αvβ3. The role of 14, 15-EET in breast cancer cell adhesion, invasion was explored by adhesion and Transwell assays. The role of 14, 15-EET in breast cancer cell cisplatin resistance in vitro was determined by MTT assay. Western blot was conducted to detect the protein expressions of EMT-related markers and FAK/PI3K/AKT signaling. Xenograft models in nude mice were established to explore the roles of 14, 15-EET in breast cancer cells EMT and cisplatin resistance in vivo.

**Results:**

In the present study, we show that serum level of 14, 15-EET increases in breast cancer patients and 14, 15-EET level of tumor tissue is higher than that of non-cancerous tissue. Moreover, 14, 15-EET increases integrin αvβ3 expression, leading to FAK activation. 14, 15-EET induces breast cancer cell EMT via integrin αvβ3 and FAK/PI3K/AKT cascade activation in vitro. Furthermore, we find that 14, 15-EET induces breast cancer cells EMT and cisplatin resistance in vivo, αvβ3 integrin and the resulting FAK/PI3K/AKT signaling pathway are responsible for 14, 15-EET induced-breast cancer cells cisplatin resistance.

**Conclusions:**

Our findings suggest that inhibition of 14, 15-EET or inactivation of integrin αvβ3/FAK/PI3K/AKT pathway could serve as a novel approach to reverse EMT and cisplatin resistance in breast cancer cells.

## Background

Breast cancer is the most common malignancies worldwide. Despite recent advances in diagnosis and treatment, it remains the second leading cause of cancer-related deaths among women [1, 2]. Chemotherapy is one of the well-established strategies of breast cancer treatment. However, drug resistance is a major cause of cancer treatment failure and cancer-related death. Therefore, it is of great clinical significance to investigate the mechanisms underlying drug resistance.

14, 15-epoxyeicosatrienoic acid (14, 15-EET) is a lipid signaling molecule which regulates various physiological processes such as proliferation, migration and inflammation [3, 4]. Recently, it has been reported that 14, 15-EET promoted tumor cell proliferation and metastasis [5–7]. Epithelial-mesenchymal transition (EMT) is the process that epithelial cells lose polarity and cell-cell adhesion, and acquire the characteristics of mesenchymal cells [8, 9]. Cells undergoing EMT display reduced expression of epithelial cell markers (such as E-cadherin, ZO-1) and increased expression of mesenchymal molecules (such as N-cadherin, vimentin, snail, slug). EMT plays a critical role in tumor cell migration, metastasis and the acquisition of stem cell-like properties [10–13]. Although the role of 14, 15-EET in tumor invasion and metastasis has been demonstrated in recent years, the mechanism underlying the role of 14, 15-EET in tumor cell EMT remains unclear.

Recently, EMT has received more and more attention for its role in cancer drug resistance. Several studies showed that the drug resistant cancer cells display features of EMT [14, 15]. It has been found that inhibition of breast cancer cell EMT could suppress cancer drug resistance [16]. These results suggested that EMT might be associated with cancer cell drug resistance. Given that 14, 15-EET promoted tumor invasion and metastasis by inducing tumor cell EMT, the role and mechanisms of 14, 15-EET in cancer cell drug resistance still remains largely unknown.

In the present study, we found that 14,15-EET induces breast cancer cell EMT, and demonstrated that 14, 15-EET up-regulates integrin αvβ3 expression, which leads to the activation of FAK/PI3K/AKT signaling. Furthermore, we revealed that integrin αvβ3 and FAK/PI3K/AKT activation is required for 14, 15-EET to induce tumor cell EMT and cisplatin resistance.

## Methods

### Patients

The study protocol was performed according to the Declaration of Helsinki and was approved by the Ethics Committee of Wuhan No.1 Hospital of Tongji Medical College. All breast cancer patients gave their signed informed consent for the use of biological samples. Tumor tissues and noncancerous tissues were collected from 11 patients in the Wuhan No.1 Hospital of Tongji Medical College.

### Cell line and animals

Human breast cancer cell MCF-7 and MDA-MB-231 were purchased from the China Center for Type Culture Collection (Wuhan, China). BALB/c athymic nude (nu/nu) mice (6–8 weeks old) were obtained from SLAC Laboratory Animal Co. Ltd. (Shanghai, China). The mice were maintained in the accredited animal facility of Tongji Medical College, and used for studies approved by the Animal Care and Use Committee of Tongji Medical College.

### Antibodies and reagents

Rabbit anti-human antibodies against integrin αv and β3, FAK, p-FAK, PI3K, p-PI3K, AKT, p-AKT, E-cadherin, N-cadherin, vimentin, snail and LY294002 (PI3K inhibitor) were purchased from Cell Signaling (Danvers, MA, USA). slug and PF562271 were purchased from Abcam (Cambridge, MA, US). The small interfering RNAs (si RNAs) against integrin αv, integrin β3 and control for experiments using targeted siRNA transfection were purchased from Santa Cruz Biotechnology (Santa Cruz, CA). Lipofectamine 2000 was purchased from Invitrogen Life Technologies (Carlsbad, CA, USA). The 14, 15-EET and 14, 15-EEZE were purchased from Cayman chemical (Ann 152 Arbor, MI, USA).

### Cell culture

MCF-7 and MDA-MB-231 cells were cultured in flasks in DMEM growth medium supplemented with 5% FBS, 100 U/ml of penicillin, and 100 pg/ml of streptomycin. The cells were cultured at 37 °C in a humidified atmosphere of 95% air and 5% CO_2_.

### Enzyme-linked immunosorbent assay (ELISA)

A stable metabolite of 14, 15-EET, 14, 15-dihydroxyeicosatrienoic acid (14, 15-DHET) in peripheral venous blood from patients with breast cancer and healthy donors or in BC tissues and noncancerous tissues from breast cancer patients was measured with ELISA (14, 15-EET/DHET ELISA kit; Detroit R&D Inc., Detroit, MI, USA) according to the manual.

### Measurement of cell proliferation

Cell viability was performed using an MTT assay. Cells were added to 96-well plates (5 × 10^3^ cells per well) following to 24 h incubation. On the following day the media were removed and the cells were treated with or without 14, 15-EET and/or 14, 15-EEZE following an incubation for 72 h. After incubation of respective time 10% of an MTT solution (2 mg/mL) was added to each well and the cells were incubated for 4 h at 37 °C. The formazan crystals that formed were dissolved in DMSO (100 μ L/well) with constant shaking for 5 min. The absorbance of the plate was then read with a microplate reader at 540 nm. Three replicate wells were evaluated for each analysis.

### Adhesion assay

Tumor cells were added to 6-well plates (5 × 10^5^ cells per well) which were pre-coated with fibronectin (Sigma). After 2 h incubation at 37 °C, non-adherent cells were harvested. Then, adherent cells were harvested by treatment with trypsin. The percentage of adherent cells was calculated. The results were expressed as A570 values. Each assay was tested in triplicate wells in three independent experiments.

### Matrigel invasion assay

Cells (5 × 10^4^) in serum-free media were seeded onto the upper chambers of modified Boyden chambers (Corning, NY, USA) in which the Transwell filter inserts were coated with Matrigel. In the lower chambers, 5% FBS was added as a chemoattractant. After incubation for 24 h, the membrane was washed briefly with PBS and fixed with 4% paraformaldehyde. The upper side of membrane was wiped gently with a cotton ball. The membrane was then stained using hematoxylin and removed. The magnitude of cells migration was evaluated by counting the migrated cells in six random clones under high-power (× 100) microscope fields. The average number of cells per field was calculated.

### Cell transfection

For silencing specific gene expression, cells were treated with integrin αv siRNA or integrin β3 siRNA. Briefly, 5 × 10^5^ MCF-7 and MDA-MB-231 cells were seeded into 6-well plate with 2 ml antibiotic-free normal growth medium containing FBS. Transfection of integrin αv siRNA, integrin β3 siRNA or control siRNA was performed according to the manufacture’s protocol.

### Quantitative RT-PCR

Total RNA was extracted from cells with TRIzol reagent (Invitrogen, Carlsbad, CA). Quantitative real-time PCR analyses were performed by Applied Biosystems using SYBR Premix Ex Taq™ (TaKaRa, Japan). The mRNA of GAPDH was used as internal control. The primers were as follows: integrin αv, sense5’-CTCGGGACTCCTGCTACCTC-3′, antisense 5’-AAGAAACATCCGGGAAGACG-3′ integrin β3, sense 5’-CCGTGACGAGATTGAGTCA-3′ antisense5’-AGGATGGACTTTCCACTAGAA-3′ and GAPDH, sense 5’-TCATTGACCTCAACTACATGGTTT-3′, antisense 5’-GAAGATGGTGATGGGATTTC-3’.The relative expression of αv and β3 was calculated using 2^-ΔΔCt^ method.

### Western blot analysis

The whole-cell extracts were prepared using RIPA lysis buffer (Beyotime, China) with phenylmethanesulfonyl fluoride and protease inhibitor cocktail (Roche, USA). Cell lysates were separated by 8–12% SDS-PAGE and electro-transferred onto polyvinylidene difluoride membranes (Bio-Rad, USA). After being blocked using 5% non-fat milk for 1 h at room temperature, membranes were incubated with the indicated primary antibodies overnight at 4 °C and probed with horseradish peroxidase-conjugated secondary antibodies (1:1000). The bands were visualized using a ChemicDocXRS system (Bio-Rad, USA).

### Immunohistochemistry

Mice were inoculated i.m. in the right hind thigh with MDA-MB-231 cells (2 × 10^6^). Tissue sections were prepared and subjected to immunohistochemical analysis. Anti-human Ki67 antibody, anti-human E-cadherin and anti-human vimentin antibody were used as primary antibodies. HRP-conjugated secondary Ab was used as secondary antibody. Images were obtained using an Olympus-IX71 microscope at 40 × 10 magnification.

For H&E staining, the tumor tissues were embedded in paraffin according to standard histological procedures. Sections were stained with hematoxylin and eosin.

### Mice xenograft models

To assay tumor cell arrest in lung during blood flow, MDA-MB-231 cells were labeled with CFSE, and injected into mice via tail vein (2 × 10^6^ cells/mouse, *n* = 8 for each group). Lungs of mice were harvested 5 h or 24 h after the injection. Frozen sections were prepared and analyzed by fluorescence microscopy. Fluorescent spots were counted from 20 randomly chosen fields in sections of each mouse.

Nude mice were inoculated i.m. in the right hind thigh with MDA-MB-231 cells(2 × 10^6^). The transplanted nude mice were randomly divided into 5 groups (*n* = 6 for each group). The mice were treated or untreated with14, 15-EET and/or 14, 15-EEZE (i.v. injection, 30 μg/kg/2d). The mice were treated with cisplatin (i.p. injection, 3.0 mg/kg/d) or PBS. All mice were examined every 2 days and sacrificed 35 days after tumor inoculation. Tumor volume (V) was monitored by measuring the length (L) and width (W) and calculated with the formula V = (L × W^2^) × 0.5.

### Statistical analysis

The values given are means ± S.E.M. The significance of difference between the experimental groups and controls was assessed by Student’s t test. The difference was significant if the *p* value was < 0.05.

## Results

### 14, 15-EET promotes breast cancer cell adhesion and migration

**Fig. 1:**
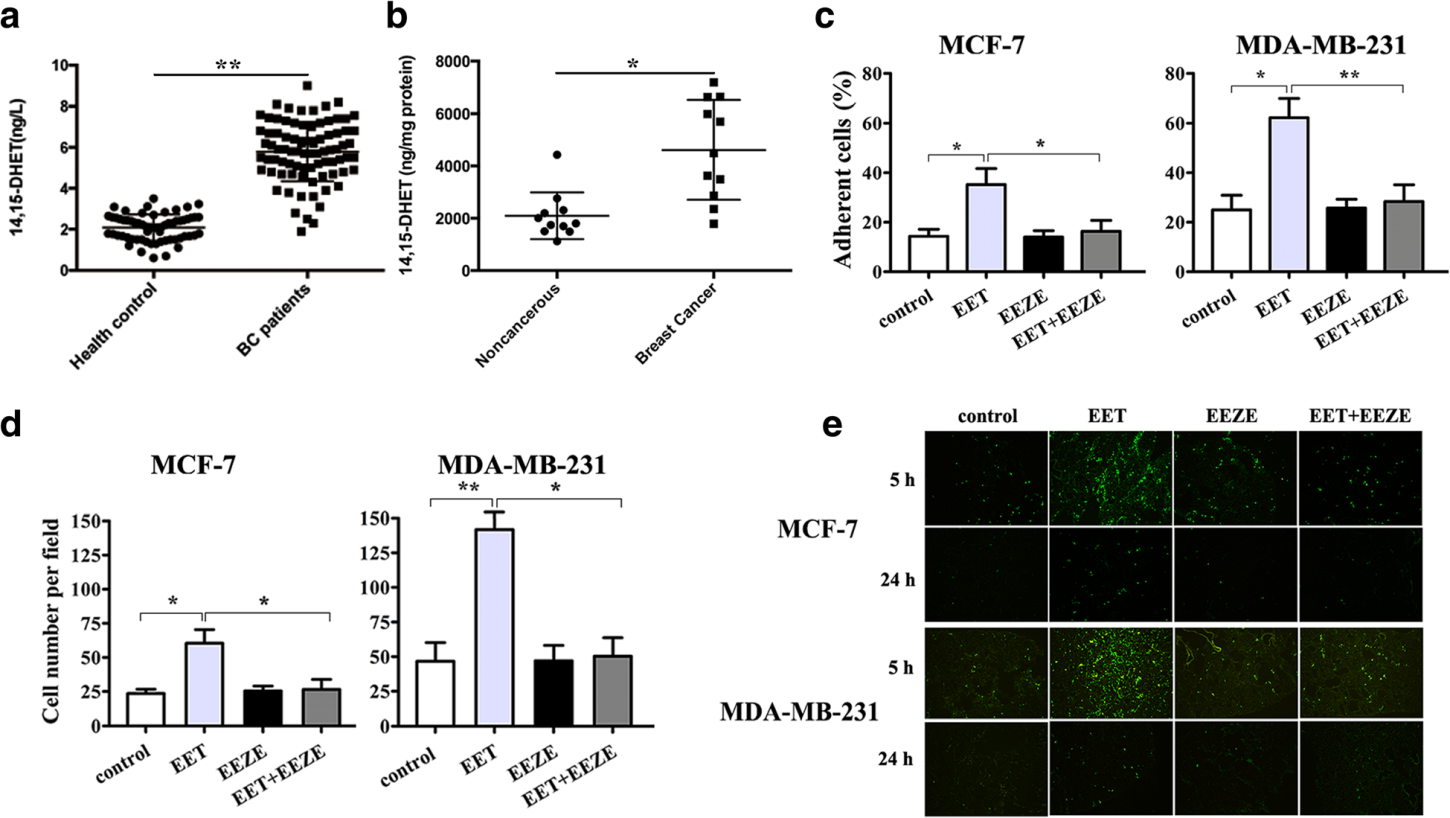
Effect of 14, 15-EET on breast cancer cell adhesion and invasion. **a** 14, 15-DHET (a stable metabolite of 14, 15-EET) level in serum of BC patients was measured by ELISA. MCF-7 and MDA-MB-231 cells were untreated or treated with 14, 15-EET (100 nM) and/or 14, 15-EEZE (200 nM). **b** Intracellular levels of 14, 15-DHET in breast cancer tissues and paired adjacent noncancerous regions. **c** The adhesion ability of tumor cells was measured by adhesion assay. **d** The invasion ability of tumor cells was measured by Matrigel invasion assay. **e** Tumor cell arrest in lung and extravasation. Tumor cells were treated or untreated with 14, 15-EET (100 nM) and/or 14, 15-EEZE (200 nM) and labeled with CFSE, and then injected to mice via tail vein. Mice were sacrificed 5 h (for analysis of tumor cell arrest) and 24 h (for analysis of extravasation) after the i.v injection of CFSE-labeled cells. The CFSE-labeled cells in frozen sections were visualized by fluorescence microscopy. Fluorescent spots in the frozen sections of lung tissues were counted. *p < 0.05, **p < 0.01

14, 15-EET has been reported to induce migration and invasion of human cancer cells [5, 6]. 14, 15-EET is very unstable metabolites, and it’s rapidly hydrolyzed by sEH to the more stable metabolites 14, 15-DHETs. We detected the 14, 15-DHET level in serum or in cancer and noncancerous tissues from breast cancer patients. The ELISA results showed that the levels of 14, 15-DHET in serum and cancer tissues in BC patients is much higher than that of healthy donors or noncancerous tissues(Fig. 1a, b). Furthermore, we found that 14, 15-EET enhanced the adhesion ability of MCF-7 and MDA-MB-231 cells (Fig. 1c). Invasion assay showed that 14, 15-EET promoted tumor cell invasion(Fig. 1d), whereas 14, 15-EEZE, an antagonist of 14, 15-EET inhibited EET-induced cell adhesion and invasion.

We further investigated the effect of 14, 15-EET on tumor cell invasion in vivo. The fluorescent spots in lung tissues were significantly increased both 5 h and 24 h after i.v. injection of MCF-7 and MDA-MB-231 cells treated with 14, 15-EET, while 14, 15-EEZE abolished the effect of 14, 15-EET on tumor cell adhesion and invasion in vivo (Fig. 1e). These results indicated that 14, 15-EET promotes breast cancer cell adhesion and extravasation.

### 14, 15-EET induces integrin αvβ3 expression and FAK/PI3K/AKT activation

**Fig. 2:**
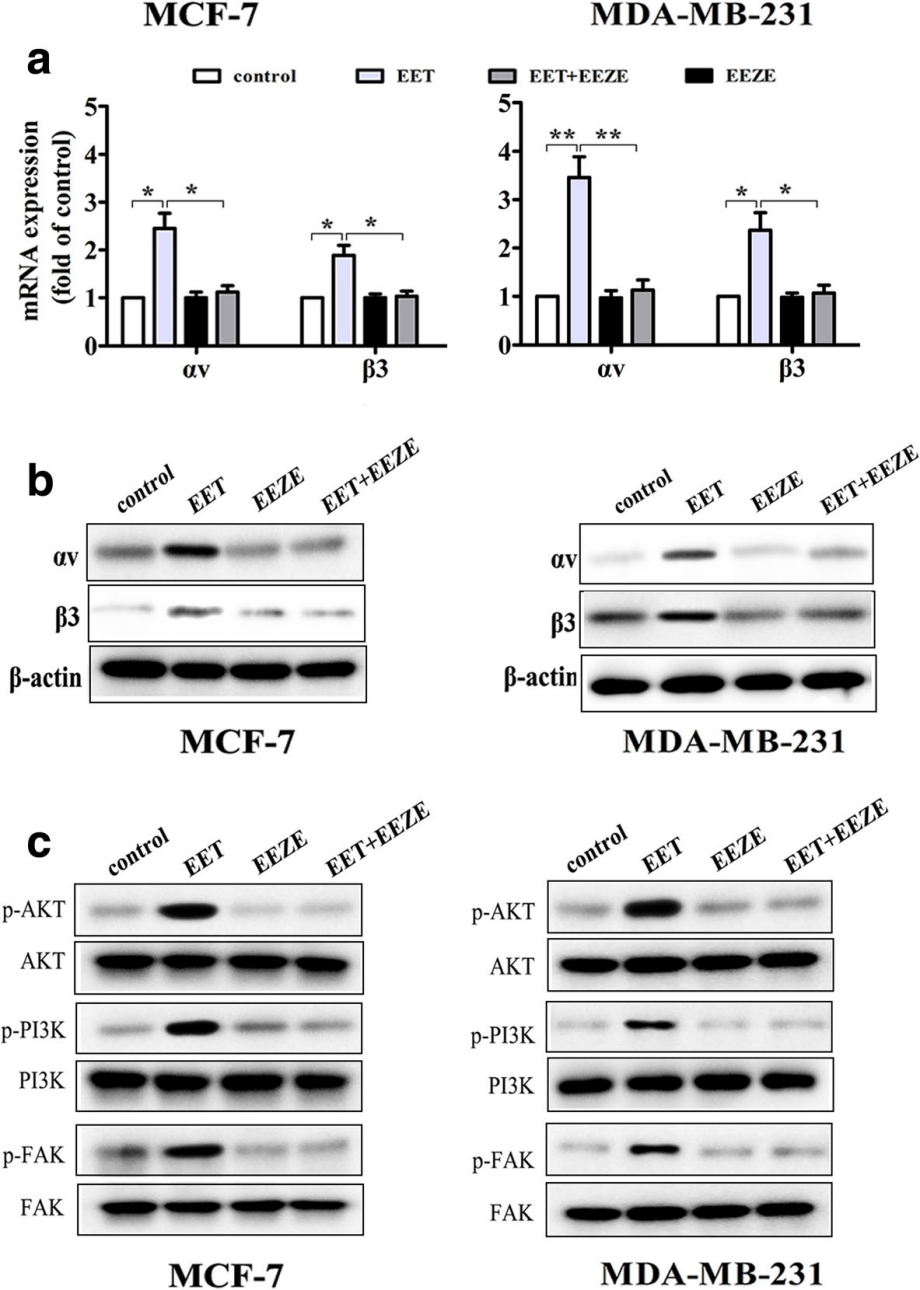
14, 15-EET up-regulates integrin αvβ3 expression and activates FAK/PI3K/AKT signaling. MCF-7 and MDA-MB-231 cells were untreated or treated with 14, 15-EET (100 nM) and/or 14, 15-EEZE (200 nM). **a** and **b** The expression of integrin αvβ3 was analyzed by real-time RT-PCR and Western blot. **c** The phosphorylated and un-phosphorylated FAK, PI3K and AKT were detected by Western blot. *p < 0.05, **p < 0.01

Fibronectin presents binding sites for a range of different integrins including integrin αvβ3. As 14, 15-EET enhanced adhesion ability of breast cancer cells to fibronectin, we hypothesized that integrin αvβ3 may be involved in 14, 15-EET-induced breast cancer cells adhesion and invasion. We found that 14, 15-EET increased the mRNA and protein expression of αv- and β3-integrin, Whereas 14, 15-EEZE, reduced EET-induced integrin αvβ3 expression (Fig. 2a, b).

It is widely known that FAK is a downstream integrin αvβ3 kinase. Since 14, 15-EET up-regulated integrin αvβ3 expression, we investigated whether 14, 15-EET affected FAK phosphorylation. We found that 14, 15-EET increased breast cancer cells FAK phosphorylation. PI3K/AKT, downstream FAK signaling molecules were also found to be activated by 14, 15-EET, while 14, 15-EEZE inhibited 14, 15-EET-induced FAK/PI3K/AKT activation (Fig. 2c).

### Integrin αvβ3 mediates 14, 15-EET-induced breast cancer cells migration and FAK/PI3K/AKT activation

**Fig. 3:**
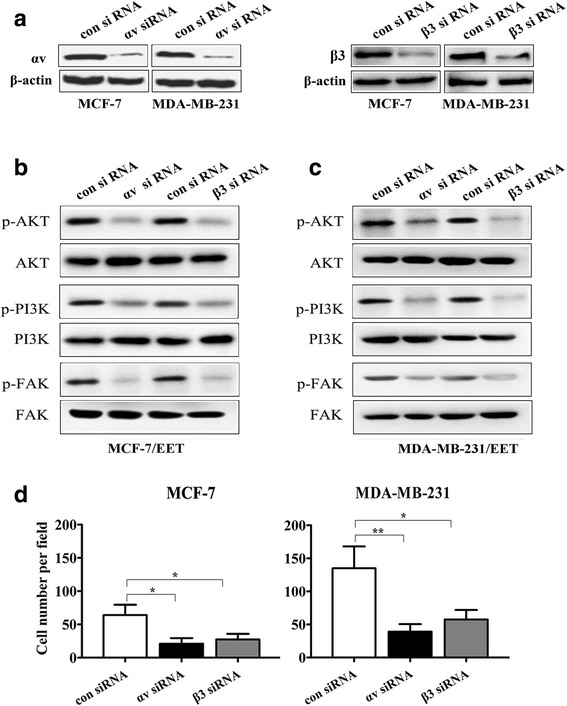
14, 15-EET promotes tumor cells invasion and activates FAK/PI3K/AKT signaling through integrin αvβ3. MCF-7 and MDA-MB-231 cells were transfected with integrin αv or β3 siRNA or control siRNA. **a** The integrin αv or β3 expression was examined by Western blot. The integrin αv or β3 knockdown tumor cells were treated with 14, 15-EET (100 nM). **b** and **c** The phosphorylated and un-phosphorylated FAK, PI3K and AKT were detected by Western blot. **d** The invasive migration assay was performed. *p < 0.05, **p < 0.01

14, 15-EET up-regulated αvβ3 integrin expression and activated FAK/PI3K/AKT signaling in breast cancer cells, therefore, we investigated whether integrin αvβ3 mediated the oncogenic effects of 14, 15-EET. Tumor cells were transfected with integrin αv or β3 siRNA, the expressions of integrin αv and β3 were validated by western blot (Fig. 3a). Knocking down of αv and β3 integrin reduced 14, 15-EET-induced tumor cell FAK/PI3K/AKT phosphorylation (Fig. 3b, c) and invasion (Fig. 3d). These results indicated that 14, 15-EET promotes breast cancer cell invasion and activates FAK/PI3K/AKT signaling through up-regulating integrin αvβ3 expression.

### Integrin αvβ3 and FAK/PI3K/AKT signaling mediate 14, 15-EET-induced breast cancer cells EMT

**Fig. 4:**
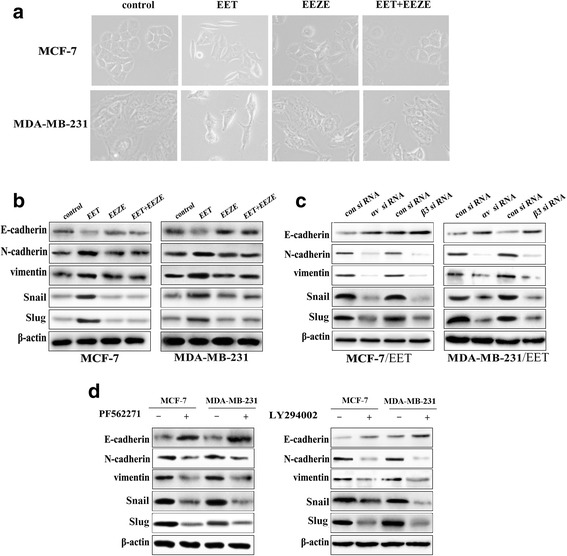
14, 15-EET induces breast cancer cells EMT via integrin αvβ3 and FAK/PI3K/AKT signaling. MCF-7 and MDA-MB-231 cells were untreated or treated with 14, 15-EET (100 nM) and/or 14, 15-EEZE (200 nM). **a** 14,15-EET induces mesenchymal morphology changes in MCF-7 and MDA-MB-231 cells: spindle shape and loss of cell-cell contact. **b** EMT markers in tumor cells were examined by Western blot. The integrin αv or β3 knockdown tumor cells were untreated or treated with 14, 15-EET (100 nM). **c** EMT markers in tumor cells were examined by Western blot. Tumor cells were untreated or treated for 30 min with PF562271 (100 nM) or LY294002 (500 nM) followed by stimulation with 14, 15-EET (100 nM). **d** EMT markers in tumor cells were examined by Western blot

The above data indicated that 14, 15-EET promoted breast cancer cell invasion, given that EMT is known to be the initial step of tumor cell migration and invasion, we examined whether 14, 15-EET affect breast cancer cells EMT. When we seeded 14,15-EET-treated MCF-7 and MDA-MB-231 cells in 6-well plate, it was found that cells lost cell-cell contact and formed a scattered phenomenon (Fig. 4a). Moreover, we found that tumor cells expressed reduced E-cadherin, increased N-cadherin, vimentin, snail and slug after treatment of 14,15-EET, while 14,15-EEZE reversed 14, 15-EET-induced EMT (Fig. 4b). We further examined whether integrin αvβ3 involved in EMT induced by 14, 15-EET. As expected, knockdown of αv or β3 integrin inhibited 14, 15-EET-induced tumor cell EMT (Fig. 4c). PI3K signaling is responsible for EMT, to further confirm the role of FAK/PI3K/AKT signaling in 14, 15-EET-induced EMT, FAK inhibitor PF562271 and PI3K inhibitor LY294002 were utilized. We found that tumor cells treated with PF562271 or LY294002 expressed high levels of E-cadherin and low levels of N-cadherin, vimentin, snail and slug compared with control cells (Fig. 4d). These results suggested that 14, 15-EET induces breast cancer cells EMT through αvβ3/FAK/PI3K/AKT signaling.

### 14, 15-EET induces breast cancer cisplatin resistance through integrin αvβ3 and FAK/PI3K/AKT signaling

**Fig. 5:**
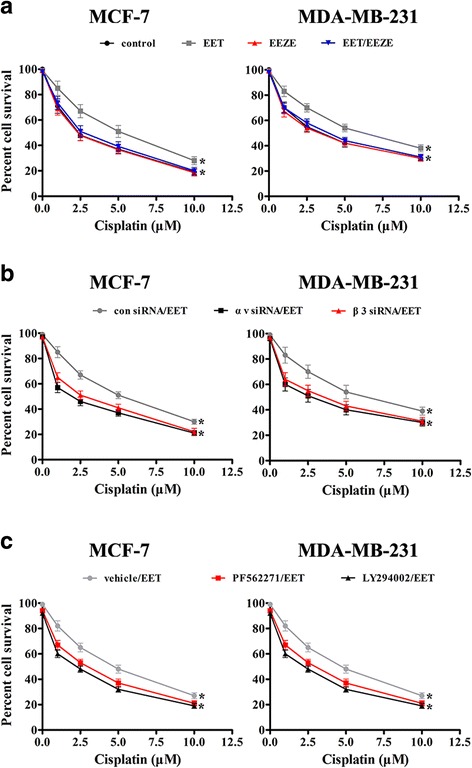
14, 15-EET induces cisplatin resistance in breast cancer cells. MCF-7 and MDA-MB-231 cells were untreated or treated with 14, 15-EET (100 nM) and/or 14, 15-EEZE (200 nM). **a** The sensitivity of tumor cells to cisplatin was determined by MTT assay. The integrin αv or β3 knockdown tumor cells were untreated or treated with 14, 15-EET (100 nM). Tumor cells were untreated or treated with PF562271 (200 nM) or LY294002 (500 nM) followed by stimulation with 14, 15-EET (100 nM). **b** and **c** The sensitivity of tumor cells to cisplatin was determined by MTT assay. *p < 0.05

Tumor cells display EMT and lead to enhanced drug resistance, as 14, 15-EET induced breast cancer cells EMT, we examined the role of 14, 15-EET in tumor cells sensitivity to cisplatin. MTT assay showed that 14, 15-EET significantly reduced tumor cells sensitivity to cisplatin, while 14, 15-EEZE reversed 14, 15-EET-induced cisplatin resistance (Fig. 5a). Knocking down of αv or β3 integrin reversed 14, 15-EET-induced tumor cells cisplatin resistance (Fig. 5b). Moreover, both PF562271 and LY294002 were found to reduce 14, 15-EET-induced tumor cells cisplatin resistance (Fig. 5c). These data indicated that integrin αvβ3 and FAK/PI3K/AKT signaling mediate 14, 15-EET-induced breast cancer cells cisplatin resistance.

### 14, 15-EET induces breast cancer cells EMT and cisplatin resistance in vivo

**Fig. 6:**
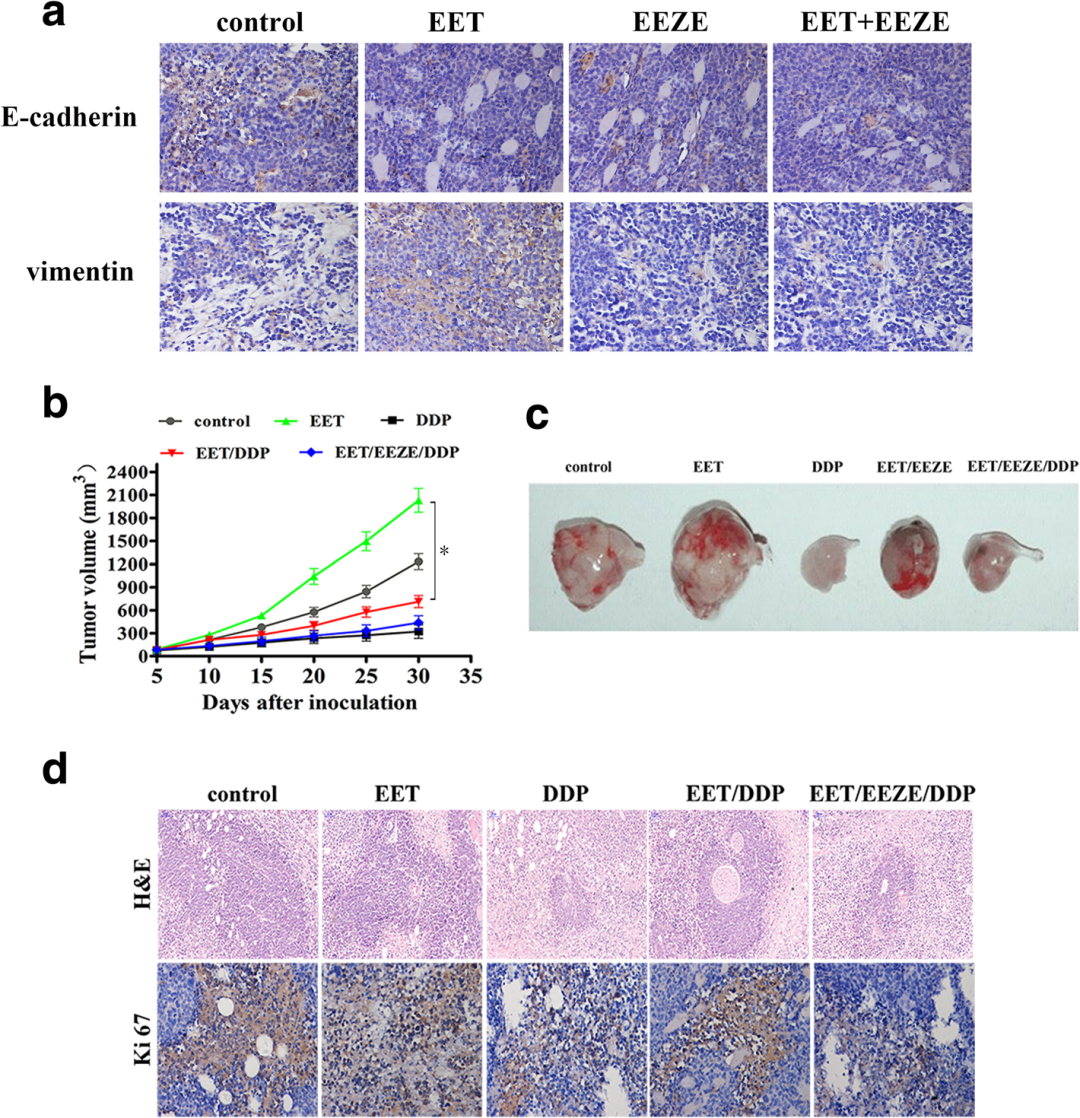
14, 15-EET induces tumor cells EMT and reduces cisplatin sensitivity of breast cancer cell in vivo*.* Nude mice were inoculated with MDA-MB-231 cells, tumors were developed in mice followed by treatment with 14, 15-EET and/or 14, 15-EEZE (i.v. injection, 30 μg/kg/2d). **a** Representative immunohistochemical staining of EMT marker. Nude mice were inoculated with MDA-MB-231 cells, tumors were developed in mice followed by treatment with 14, 15-EET and/or 14, 15-EEZE (i.v. injection, 30 μg/kg/2d). All mice were treated with cisplatin (i.p. injection, 3.0 mg/kg/d) or PBS. **b** The gross morphology of tumor samples. **c** The tumors volume was measured on the indicated days. **d** Tumors from mouse xenografts were removed and subjected to H&E staining and immunohistochemistry for Ki67. *p < 0.05

We further evaluated the role of 14, 15-EET on MDA-MB-231 cells EMT and cisplatin resistance in xenograft model. In line with our earlier observations, the expression of E-cadherin decreased while the expression of vimentin increased obviously after 14,15-EET treament. (Fig. 6a). The average tumor volume of cisplatin-treated tumors was significantly smaller than that of 14, 15-EET/cisplatin-treated tumors (Fig. 6b, c). Histologic examination of the tumors showed dramatically increased cellularity in the 14, 15-EET/cisplatin-treated compared with cisplatin-treated tumors (Fig. 6d). Immunohistochemical staining of the 14, 15-EET/cisplatin-treated compared with cisplatin-treated tumors showed increased Ki67 levels (Fig. 6d). Whereas 14, 15-EEZE, reversed 14, 15-EET’s effect. These results suggested that 14, 15-EET promotes breast cancer cells EMT and reduces cisplatin sensitivity in vivo.

## Discussion

To develop a novel and efficient therapy for human breast cancer treatment, it is necessary to elucidate the molecular mechanisms underlying tumor metastasis and drug resistance. Accumulating evidence have suggested that 14, 15-EET promotes tumor metastasis and progression in various cancers including breast cancer [17, 18]. In the present study, we demonstrated that 14, 15-EET up-regulates integrin αvβ3 expression and results in FAK/PI3K/AKT activation. Furthermore, we found that 14, 15-EET induces breast cancer cells EMT and cisplatin resistance through integrin αvβ3 and its downstream FAK/PI3K/AKT/ signaling. Our finding provide an insight into the function of 14, 15-EET in regulating breast cancer cell EMT and cisplatin resistance.

EET has been reported to enhance tumor cell motility, invasion and metastasis [7, 19]. Our previous study found that 14, 15-EET induced neutrophils infiltration and promoted tumor metastases [17]. EMT is associated with tumor invasive and metastatic potential. However, the relationship between 14, 15-EET and breast cancer cell EMT has not been investigated. Our current study provide evidence that 14,15-EET induced breast cancer cells EMT, as demonstrated by the changed levels of EMT markers and cell morphology.

Recently, the molecular mechanisms of EMT have been extensively investigated, several signaling pathways that induce EMT have been discovered [20–22]. Integrin αvβ3 has been shown to be frequently implicated in the metastasis of various tumor types [23–25]. It has been reported that integrin αvβ3 is involved in tumor cell EMT [26–28]. In the current study, we found that 14, 15-EET led to a significant increase in mRNA and protein level of integrins αv and β3. In contrast, treatment of its antagonist 14, 15-EEZE resulted in a reversal of the 14, 15-EET effects on integrin αvβ3 expression. To understand the mechanism of 14,15-EET-induced EMT, we silenced the breast cancer cells integrin αvβ3. We found knockdown of integrin αv and β3 reversed the effects of 14, 15-EET on the levels of EMT markers and cell morphology, these findings further confirm that integrin αvβ3 mediates breast cancer cells EMT induced by 14,15-EET.

Integrin signaling is depending on the formation of adhesion complexes including FAK, after activation of FAK by integrins, activated FAK phosphorylates the downstream PI3K and then activates Akt [29]. Our previous study found that integrin αvβ3 activated FAK and promoted tumor invasion [23]. Several studies have reported the role of FAK signaling in the induction of EMT [30, 31]. 14, 15-EET has been reported to activate PI3K/AKT signaling [32]. To further elucidate the molecular mechanism of 14,15-EET-induced EMT we focused on signaling pathway implicating FAK and the downstream PI3K/AKT signaling. We demonstrated that 14, 15-EET activates breast cancer cells FAK/PI3K/AKT signaling through up-regulating integrin αvβ3. Furthermore, we found that inhibiting FAK by a pharmacological inhibitor of FAK, PF-562271 reversed the EMT induced by 14,15-EET. Similar study was reported that FAK play a crucial role EMT through the activation of Akt signaling pathway [30]. A recent study demonstrated that FAK/PI3K/AKT signaling mediates the hepatocellular carcinoma EMT [26]. In our study, we found that PI3K/AKT signaling inhibitor, LY294002 abrogate 14,15-EET-induced breast cancer cells EMT.

It is becoming increasingly evident that EMT is frequently accompanied with cancer drug resistance in various cancer [33–35]. Inhibition EMT reversed the drug resistance [36, 37]. It has been reported that integrin β3 mediated tumor cell erlotinib resistance [38]. In our present study, we demonstrated that 14, 15-EET enhanced breast cancer cisplatin resistance, knockdown of integrin αv and β3 abolished 14, 15-EET-induced cisplatin resistance. PI3K/AKT signaling activation may be involved in tumor cell resistance to sorafenib [26]. It has been reported that activation of AKT signaling led to drug resistance in breast cancer and ovarian cancer [39, 40]. Moreover, AKT was found to mediate the Twist2-induced cisplatin resistance [41]. Here, we found that knockdown of integrin αv and β3 partially inhibits FAK/PI3K/AKT activation in 14, 15-EET-treated tumor cells. As expected, inhibition of FAK and PI3K/AKT activation by PF562271 or LY294002 substantially sensitized tumor cells to cisplatin.

As outlined above, both integrin αvβ3 and FAK/PI3K/AKT signaling have been involved in 14, 15-EET-induced breast cancer cells EMT and cisplatin resistance. Further study will be needed to refine our mechanistic model. The data on the intermediate factors connecting 14, 15-EET to integrin αvβ3 is little. In addition, knockdown of integrin αv and β3 only partially inhibited the activation of FAK/PI3K/AKT, suggesting that additional factors downstream of 14, 15-EET may also lead to 14, 15-EET-induced EMT and cisplatin resistance.

## Conclusions

**Fig. 7:**
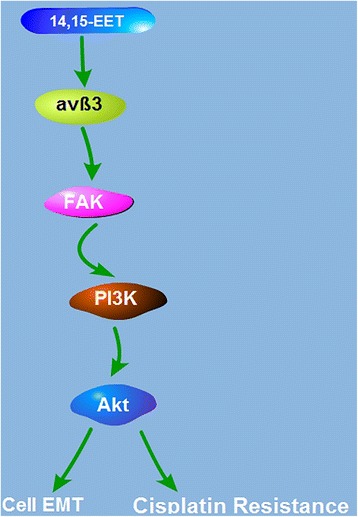
Proposed schematic model for EMT and cisplatin resistance induced by 14, 15-EET in breast cancer cells. 14, 15-EET up-regulates integrin αvβ3 and activates FAK/PI3K/AKT signaling, ultimately resulting in breast cancer cells EMT and cisplatin resistance

Taken together, we define a non-canonical function for 14, 15-EET as an inducer of breast cancer cells EMT and drug resistance. Our results show that 14, 15-EET activates the FAK/PI3K/AKT pathway by up-regulating expression of αvβ3 integrin, leading to enhanced breast cancer cells EMT and cisplatin resistance (Fig.7). Our study suggests that targeting 14, 15-EET or integrin αvβ3 signaling will be able to reverse breast cancer cells EMT and cisplatin resistance.

## Abbreviations

14, 15-DHET: 14, 15-dihydroxyeicosatrienoic acid
14, 15-DHET: 14,15-dihydroxyeicosatrienoic acid
14, 15-EET: 14, 15-epoxyeicosatrienoic acid
CFSE: Carboxyfluorescein diacetate, succinimidyl ester
DMSO: Dimethyl sulfoxide
EMT: Epithelial-mesenchymal transition
FAK: Focal adhesion kinase
FN: fibronectin
Ki67: Nuclear-associated antigen
MTT: 3-(4, 5-Dimethylthiazol-2-yl)-2, 5-diphenyltetrazolium bromide
PI3K: phosphatidylinositol 3 kinase
SDS-PAGE: Sodium dodecyl sulfate polyacrylamide gel electrophoresis
siRNAs: small interfering RNAs
